# The Role of Epicardial Fat Thickness and B-type Natriuretic Peptide (BNP)/N-terminal Pro B-type Natriuretic Peptide (NT-proBNP) in Heart Failure Risk Stratification: A Systematic Review

**DOI:** 10.7759/cureus.84184

**Published:** 2025-05-15

**Authors:** Areij Awad Osman Mohamed, Sahla Shurahbeel Bashir Omer, Musab Mukhtar, Tagwa Mohmmed, Wadah Mohamed Hussein Mohamed, Miska Haroun Mohamed Hassan, Ibrahim Mohammed Hassan

**Affiliations:** 1 Cardiology, Sudan Heart Center, Khartoum, SDN; 2 Cardiology, Sheikh Khalifa Specialty Hospital, Ras Al-Khaimah, ARE; 3 Critical Cardiac Care, Sheikh Khalifa Specialty Hospital, Ras Al-Khaimah, ARE; 4 Gastroenterology, Aberdeen Royal Infirmary, Aberdeen, GBR; 5 Internal Medicine, Sligo General Hospital, Sligo, IRL; 6 General Practice, Sukoon International Extended Center, Jeddah, SAU; 7 Cardiology, Prince Sultan Cardiac Center, Najran, SAU

**Keywords:** bnp, epicardial fat thickness, heart failure, nt-probnp, risk stratification

## Abstract

Heart failure (HF) remains a global health challenge, necessitating improved risk stratification tools. This systematic review evaluates the combined role of epicardial fat thickness (EFT) and B-type natriuretic peptide (BNP)/N-terminal pro B-type natriuretic peptide (NT-proBNP) in HF risk stratification, examining their pathophysiological interplay and clinical utility across diverse populations, including a wide age range, various comorbidities (e.g., obesity, diabetes, and systemic sclerosis), and geographic regions. EFT, a measurable marker of epicardial adipose tissue (EAT) located between the myocardium and visceral pericardium, was evaluated alongside BNP/NT-proBNP, established biomarkers of cardiac stress. Following the Preferred Reporting Items for Systematic Reviews and Meta-Analyses 2020 guidelines, 12 case-control studies were included after screening 192 records from PubMed/MEDLINE, Embase, Scopus, Web of Science, and Cochrane Library. Studies assessed EFT and BNP/NT-proBNP in HF or at-risk populations. Methodological quality was appraised using the Newcastle-Ottawa Scale (NOS). EFT consistently correlated with elevated BNP/NT-proBNP, though patterns differed by HF phenotype. In HF with reduced ejection fraction (HFrEF), NT-proBNP associated more strongly with muscle loss than adiposity, while in HF with preserved ejection fraction (HFpEF), EFT was linked to metabolic comorbidities and inflammatory markers. Paradoxically, lower EFT predicted worse outcomes in nonischemic cardiomyopathy (NICMP), potentially reflecting disease-related fat depletion or cachexia; this finding underscores the need for phenotype-specific interpretation of EFT in risk stratification. Mechanistically, EAT contributed to myocardial remodeling via adipokine secretion and inflammatory signaling. Four studies had a low risk of bias (NOS ≥ 8), while one showed a high risk. The combined assessment of EFT and BNP/NT-proBNP offers complementary prognostic insights, EFT capturing subclinical inflammation and adiposity-related remodeling, while BNP/NT-proBNP reflects myocyte stress, potentially guiding personalized treatment decisions, including closer monitoring of HFpEF patients with elevated EFT and early nutritional or anti-inflammatory interventions in those with muscle loss and elevated NT-proBNP. Inclusion criteria encompassed adult populations with HF or related conditions, with exclusion of reviews, case reports, and non-English articles, supporting the methodological rigor of this synthesis. Standardized EFT measurement and targeted EAT-modulating therapies warrant further investigation.

## Introduction and background

Heart failure (HF) continues to pose a major global health burden, affecting over 64 million people worldwide and accounting for substantial morbidity, mortality, and healthcare expenditure [1]. Despite advances in therapeutics and diagnostics, the clinical management of HF remains complex, owing to its heterogeneous presentation, progressive nature, and multifactorial etiology. Timely and accurate risk stratification is critical for optimizing therapeutic decisions, anticipating clinical deterioration, and improving long-term outcomes in affected patients [2]. In this context, the quest for reliable, noninvasive biomarkers and imaging parameters that can refine current risk assessment models is both urgent and ongoing [3].

Epicardial fat thickness (EFT), an emerging imaging biomarker, has attracted significant scientific attention due to its unique anatomical and metabolic characteristics [4]. Located between the myocardium and visceral layer of the pericardium, epicardial adipose tissue (EAT) is not merely an inert fat depot but an active endocrine organ [5]. It secretes a wide array of proinflammatory and proatherogenic cytokines that directly influence the adjacent myocardium and coronary arteries through paracrine and vasocrine mechanisms. An increased EFT has been associated with myocardial remodeling, diastolic dysfunction, and adverse cardiovascular outcomes, suggesting its potential role in the pathophysiological continuum of HF [6].

Parallel to this, B-type natriuretic peptide (BNP) and its inactive N-terminal fragment (NT-proBNP) have long been established as pivotal biomarkers in the diagnosis and prognosis of HF [7]. Secreted by cardiac myocytes in response to ventricular stretch and wall stress, BNP and NT-proBNP levels reflect cardiac load and remodeling processes [8]. These biomarkers are embedded in clinical guidelines as essential tools not only for diagnostic clarity but also for risk stratification, therapeutic monitoring, and prognostication [9].

HF is broadly categorized into HF with reduced ejection fraction (HFrEF) and HF with preserved ejection fraction (HFpEF), each with distinct pathophysiological underpinnings [3]. HFrEF is characterized by systolic dysfunction and neurohormonal activation, whereas HFpEF involves diastolic dysfunction and is closely linked to systemic inflammation and metabolic comorbidities. These differing mechanisms may influence how biomarkers such as EFT and BNP/NT-proBNP behave and interact [7].

The intersection between structural parameters such as EFT and biochemical markers like BNP/NT-proBNP presents a promising frontier in cardiovascular research. While BNP/NT-proBNP provides dynamic insights into cardiac stress and fluid overload, EFT may offer complementary information regarding subclinical inflammation and cardiac adiposity, a factor increasingly implicated in HF progression [10]. Combining EFT with BNP/NT-proBNP may thus yield additive or synergistic prognostic value, particularly by capturing distinct aspects of HF pathogenesis (mechanical, hormonal, and inflammatory). Recent studies have explored this integrative approach, proposing that the combined assessment of EFT and natriuretic peptides could enhance risk stratification and individualize patient care more effectively than either parameter alone [4].

However, despite growing interest and numerous individual studies, the collective evidence on the role of EFT in conjunction with BNP/NT-proBNP for HF risk stratification remains fragmented and inconclusive. Uncertainties persist regarding clinically meaningful EFT thresholds, population-specific cutoffs, and the phenotype-specific utility of these markers, particularly in HFpEF vs. HFrEF. Variations in study designs, populations, measurement techniques, and endpoints have contributed to heterogeneity in findings. Therefore, a systematic synthesis of current literature is warranted to clarify the prognostic utility of these parameters, identify gaps in knowledge, and inform future clinical and research directions.

This systematic review aims to assess the correlation strength between EFT and BNP/NT-proBNP levels in HF and at-risk populations, evaluate how this relationship varies by HF phenotype (HFrEF vs. HFpEF), determine their combined prognostic accuracy in HF risk stratification, and identify methodological limitations and underexplored areas to guide future research. By critically appraising and integrating data across diverse studies, we seek to elucidate their individual and combined prognostic significance, examine methodological consistencies and inconsistencies, and assess their potential as synergistic tools in the personalized management of HF.

## Review

Methodology

Protocol and Registration

This systematic review was conducted in accordance with the Preferred Reporting Items for Systematic Reviews and Meta-Analyses (PRISMA) 2020 guidelines [11]. A detailed review protocol outlining the objectives, inclusion criteria, data extraction procedures, and analysis plan was developed before the initiation of the study. However, the protocol was not registered on a public platform. We acknowledge that a lack of registration may limit transparency and raise concerns about potential selective reporting. The decision not to register was based on the team’s intention for rapid evidence synthesis.

Eligibility Criteria

Studies were eligible for inclusion if they assessed the role of EFT and/or BNP or NT-proBNP in the risk stratification of HF in adult human populations. We considered both prospective and retrospective observational studies, cross-sectional studies, and cohort studies. Interventional studies were included only if they reported baseline EFT and/or natriuretic peptide levels in the context of risk stratification. Case reports, editorials, reviews, and conference abstracts without full data were excluded. Gray literature, such as theses, preprints, or non-peer-reviewed articles, was not included, which may limit comprehensiveness and introduce publication bias. Only studies published in English and in peer-reviewed journals were considered.

Information Sources and Search Strategy

A comprehensive literature search was conducted across multiple electronic databases, including PubMed/MEDLINE, EMBASE, Scopus, Web of Science, and the Cochrane Library from their earliest available records through March 23, 2025. Additionally, reference lists of included studies were also screened to identify potentially relevant articles. The search strategy was formulated using a combination of Medical Subject Headings terms and free-text keywords related to "epicardial fat thickness", "epicardial adipose tissue", "BNP", "NT-proBNP", "heart failure", and "risk stratification". Boolean operators (AND, OR) and truncations were applied to refine the search, and the strategy was adapted for each database accordingly. The detailed search strings for each database are provided in the Appendix.

Study Selection

All identified records were imported into EndNote X9 (Clarivate Analytics, Philadelphia, PA), a reference management software, and duplicates were removed. Two independent reviewers (WMHM and IMH) conducted the title and abstract screening based on the predefined eligibility criteria. Full-text articles were subsequently retrieved for studies deemed potentially eligible. The same reviewers independently assessed the full texts for inclusion. Discrepancies at any stage were resolved through discussion or consultation with a third reviewer (TM) to reach consensus. Interrater agreement was assessed using Cohen’s kappa statistic to evaluate consistency during study selection. The study selection process was documented and presented using the PRISMA 2020 flow diagram.

Data Extraction

A standardized data extraction form was developed and pilot-tested to ensure consistency and comprehensiveness. Two reviewers independently extracted data from the included studies, recording information on study characteristics (authors, year of publication, country, and study design), population demographics (sample size, age, and sex distribution), HF characteristics (type and severity), methods of measuring EFT and BNP/NT-proBNP, primary outcomes related to risk stratification, and key findings. Any disagreements during data extraction were resolved through mutual discussion or the involvement of a third reviewer. Interrater agreement was also evaluated during data extraction to ensure consistency.

Risk of Bias Assessment

The methodological quality of the included studies was independently assessed by two reviewers using the Newcastle-Ottawa Scale (NOS) [12] for observational and case-control studies. This tool evaluates the risk of bias across three domains: selection of participants, comparability of study groups, and assessment of outcomes. Studies were rated as low, moderate, or high risk of bias based on their cumulative scores. Any discrepancies in quality assessment were resolved by consensus or adjudication by a third reviewer.

Data Synthesis

Due to the expected heterogeneity in study populations, methodologies, and outcome measures, a narrative synthesis approach was employed. The findings were grouped and summarized thematically according to the roles of EFT and BNP/NT-proBNP in HF risk stratification. Particular attention was given to studies that evaluated both markers concurrently. Where sufficient homogeneity was defined as studies using comparable outcome measures (e.g., mortality and New York Heart Association, NYHA, class), similar biomarker assessment methods, and consistent HF phenotypes, meta-analytic pooling was considered using random-effects models. However, the decision to conduct a meta-analysis was contingent upon the availability of comparable data across studies.

Reporting and Ethical Considerations

This review was conducted using publicly available published data and did not involve human participants or require ethical approval. The results are presented in adherence with the PRISMA checklist, ensuring full transparency in reporting and methodology.

Results

Studies' Selection Process

**Figure 1:**
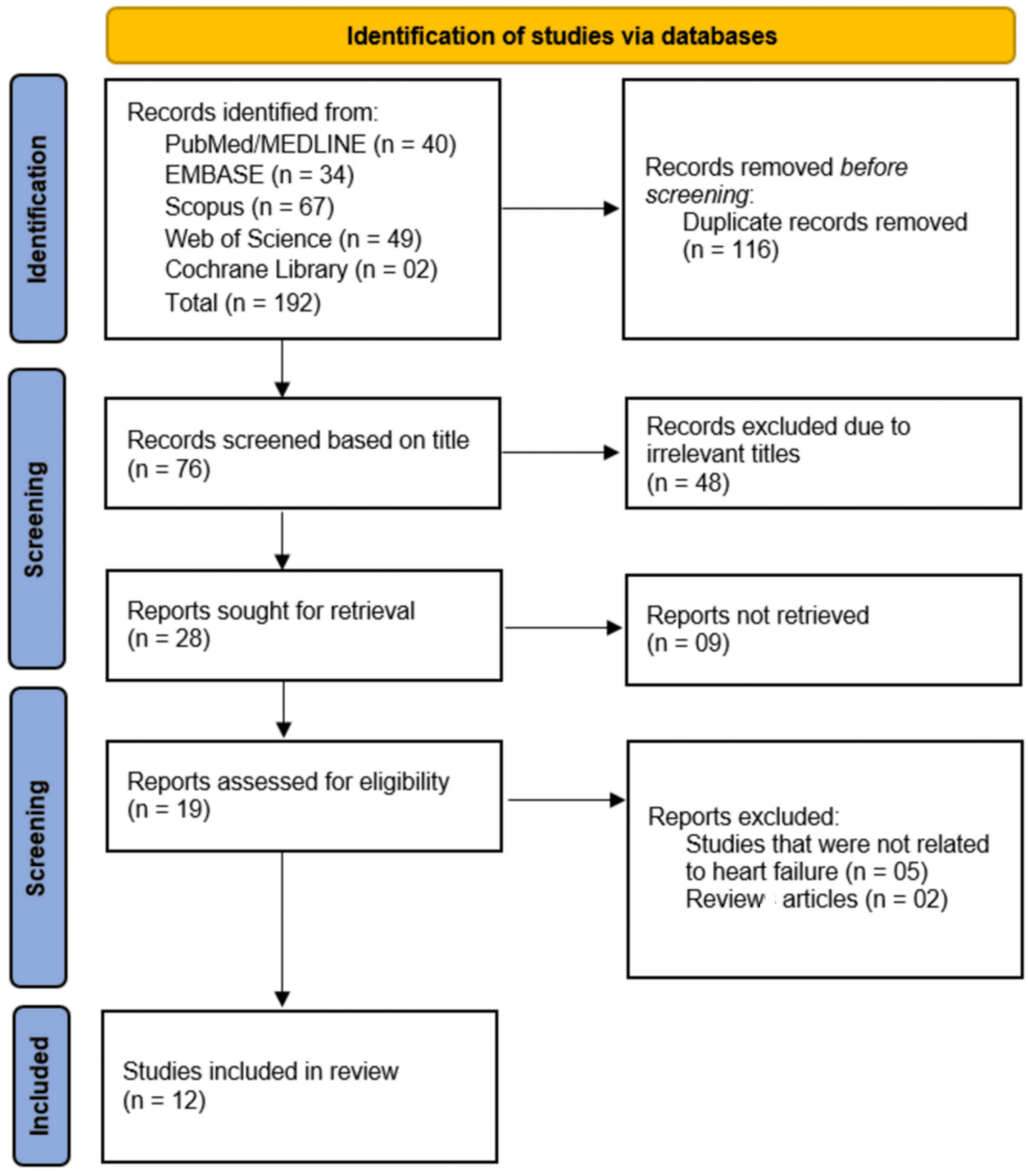
PRISMA flowchart of study selection process PRISMA: Preferred Reporting Items for Systematic Reviews and Meta-Analyses Image credit: This is an original image created by the author Musab Mukhtar

A total of 192 records were identified from databases including PubMed/MEDLINE (n = 40), EMBASE (n = 34), Scopus (n = 67), Web of Science (n = 49), and Cochrane Library (n = 2). After 116 duplicate records were removed, 76 studies were screened based on title, of which 48 were excluded for irrelevance. The remaining 28 reports were sought for retrieval, but nine could not be retrieved due to paywall restrictions. Of the 19 reports assessed for eligibility, five were excluded for not being related to HF, and two were review articles, leaving 12 studies included in the final review (Figure 1).

Characteristics of Included Studies

The systematic review included 12 studies [13-24] published between 2010 and 2021, comprising case-control, cross-sectional, and clinical-experimental designs. Sample sizes ranged from 23 to 572 participants, with populations including HF patients (HFrEF, HFpEF, and HF with mildly reduced ejection fraction), stable coronary artery disease (CAD), obesity, Cushing’s syndrome, systemic sclerosis, and stroke patients. EFT was assessed primarily via echocardiography, cardiac magnetic resonance (CMR), and biopsy. BNP/NT-proBNP levels were measured in all studies, with correlations examined between EFT, biomarkers, and clinical outcomes (Table 1).

**Table 1:** Summary of studies included in this review EFT: epicardial fat thickness, EAT: epicardial adipose tissue, BNP: B-type natriuretic peptide, NT-proBNP: N-terminal pro B-type natriuretic peptide, IMT: intima-media thickness, TDI: tissue Doppler imaging, IRT: isovolumic relaxation time, ICT: isovolumic contraction time, MPI: myocardial performance index, LV: left ventricle, LVEF: left ventricular ejection fraction, NYHA: New York Heart Association, TTE: transthoracic echocardiography, hs-CRP: high-sensitivity C-reactive protein, hs-cTnT: high-sensitivity cardiac troponin T, CAD: coronary artery disease, NICMP: nonischemic cardiomyopathy, SSc: systemic sclerosis, HF: heart failure, HFrEF: heart failure with reduced ejection fraction, HFpEF: heart failure with preserved ejection fraction, HFmrEF: heart failure with mildly reduced ejection fraction, CMR: cardiac magnetic resonance, AF: atrial fibrillation, T2DM: type 2 diabetes mellitus, CK-MB: creatine kinase-MB, FABP3: fatty acid binding protein 3, PPARα: peroxisome proliferator-activated receptor alpha, IL-6: interleukin 6, CYP11B2: aldosterone synthase, MAPK: mitogen-activated protein kinase, CTRP1: C1q/TNF-related protein 1, SAT: subcutaneous adipose tissue, RT-PCR: reverse transcription polymerase chain reaction, ELISA: enzyme-linked immunosorbent assay, CHF: congestive heart failure, HOMA-IR: homeostasis model assessment for insulin resistance

Study	Country	Study design	Sample size	Population characteristics	Heart failure type	EFT assessment method	BNP/NT-proBNP assessment	Key outcomes measured	Main findings
Bassareo et al. [13]	Italy	Case-control	23	Adolescent girls (11-17 years; mean age, 14.3 ± 1.7 years) with Cushing’s syndrome, assessed before and after surgical cure	Not specified (focus on early cardiovascular dysfunction)	EFT via echocardiogram	NT-proBNP measured (method not specified)	EFT, IMT, NT-proBNP, TDI parameters (IRT, ICT, and MPI)	EFT, IMT, NT-proBNP, and TDI parameters are altered in CS patients even after surgery compared to controls; NT-proBNP showed significant correlation with diastolic dysfunction markers and may be the best early marker of CV dysfunction in pediatric CS
Saritas et al. [14]	Turkey	Case-control	70	50 obese children and 20 nonobese children (control group with innocent murmur)	Not specified (asymptomatic)	Transthoracic echocardiography (echocardiogram)	Serum NT-proBNP levels measured during evaluation	Left ventricular systolic function, LV mass index, myocardial tissue rates, MPI, EAT thickness, carotid intima-media thickness, and NT-proBNP levels	Obese children had higher EAT, carotid IMT, and NT-proBNP levels. No significant correlation between cardiac function and NT-proBNP, carotid IMT, or EAT. Routine measurement not indicated in asymptomatic obese children
Börekçi et al. [15]	Turkey	Cross-sectional	439	Stable CAD patients undergoing coronary angiography; mean age: 62.2 ± 10.7 years	Not specified (CAD population; HF not explicitly studied)	Two-dimensional echocardiography	NT-proBNP measured	SYNTAX score, NT-proBNP, hs-CRP, hs-cTnT, and uric acid levels	Higher EFT associated with increased NT-proBNP, hs-CRP, hs-cTnT, uric acid, diabetes, and CAD complexity (SYNTAX score); EFT independently predicted NT-proBNP levels
Tabakci et al. [16]	Turkey	Case-control	131 total (93 NICMP patients, 38 healthy controls)	NICMP patients and age- and sex-matched healthy individuals	NICMP	Echocardiography: measured during end-systole on the free wall of the right ventricle	BNP levels measured in NICMP patients	EFT, EFT/BMI, LVEF, BNP levels, NYHA class, and left atrial volume index	NICMP patients had lower EFT than controls; EFT and EFT/BMI correlated with BNP and LVEF; lower EFT associated with more severe NYHA class; EFT independently associated with impaired functional status
Altun et al. [17]	Turkey	Case-control	143 (61 stroke patients, 82 controls)	Adults (≥18 years) with first acute ischemic stroke vs. matched controls	Not specified	TTE	NT-proBNP measured from blood samples at admission	Epicardial fat thickness, NT-proBNP levels, and aortic stiffness indices	Stroke patients had significantly higher EFT, lower aortic distensibility, and strain. EFT correlated with NT-proBNP and arterial dysfunction. EFT may indicate subclinical organ damage
Temiz Karadag et al. [18]	Turkey	Case-control	47 SSc patients; 36 controls	Patients with SSc without overt cardiac disease	Not specified	Transthoracic conventional Doppler echocardiography	BNP measured	EAT thickness, BNP levels, inflammatory markers, insulin resistance (HOMA-IR), and lipid profile	EAT thickness significantly higher in SSc vs. controls; correlated with age, ESR, CRP, insulin, HbA1c, and total and LDL cholesterol. BNP also elevated in SSc
Karayannis et al. [19]	Greece	Case-control	Not stated	Patients with noncachectic HF and age- and sex-matched healthy controls	Not clearly stated (likely mixed or not specified)	Echocardiography	BNP measured	Epicardial fat thickness, BMI, plasma leptin, ghrelin, adiponectin, hsCRP, and BNP	EFT positively correlated with BMI in both groups; negatively related with BNP and positively with log leptin in HF group. In controls, EFT correlated with hsCRP (positive) and log ghrelin (negative). Leptin predicted EFT in HF but not after BMI adjustment; ghrelin and hsCRP remained significant in controls even after BMI adjustment
Fosshaug et al. [20]	Norway	Case-control	60	30 patients with systolic HF and 30 with normal systolic function undergoing thoracic surgery	Systolic HF	Biopsy of EAT and SAT; analysis via RT-PCR and gas chromatography	NT-proBNP measured from plasma samples	mRNA expression of inflammatory and metabolic mediators in adipose tissue; FA composition; correlation with NT-proBNP and LVEDD	HF patients had higher mRNA expression (IL-6, adrenomedullin, PPARα, and FABP3) and higher levels of palmitoleic acid in EAT; palmitoleic acid correlated with NT-proBNP and LVEDD; EAT implicated in myocardial remodeling via endocrine action
Yang et al. [21]	China	Case-control	Not specified	Subjects with and without CHF	CHF	EAT sample analysis	BNP assessment (role inferred in pathway, not direct measurement)	CTRP1 levels in plasma and EAT, IL-6 mRNA, CYP11B2, and MAPK pathway activation	CTRP1 levels were higher in CHF patients’ plasma and EAT; CTRP1 involved in CHF pathogenesis via IL-6 and aldosterone modulation; high CTRP1 levels linked to worse prognosis
Agra et al. [22]	Spain	Case-control	74 clinical study and five experimental	Patients admitted with de novo HF	De novo HF	Adiponectin levels via ELISA and PCR in adipose cells	proBNP levels measured (method not specified)	Death or all-cause readmission, and adiponectin expression	Higher adiponectin and proBNP linked to worse nutritional status; only adiponectin independently predicted outcomes. Nutrient starvation increased adiponectin expression in adipose cells
Van Woerden et al. [23]	The Netherlands	Case-control	84 (64 HF patients, 20 controls)	HF patients with LVEF >40%, controls matched by age, sex, and BMI	HFpEF and HFmrEF	CMR: epicardial fat quantified on short-axis cine stacks	Not reported	Epicardial fat volume, comorbidities (AF and T2DM), cardiac biomarkers (CK-MB, troponin T, and HbA1c), and cardiac structure/function	HF patients had significantly more epicardial fat than controls; epicardial fat correlated with AF, T2DM, and biomarkers of myocardial injury (CK-MB, troponin T, and HbA1c)
Selvaraj et al. [24]	United States	Case-control	572	Adults without HF (n = 367), with HFrEF (n = 113), or HFpEF (n = 92)	HFrEF and HFpEF	Cardiac magnetic resonance imaging	NT-proBNP measured in 334 participants	Body composition, axial skeletal muscle mass, fat distribution, NT-proBNP levels, and all-cause mortality	Lower axial skeletal muscle size (not fat) predicts mortality. NT-proBNP is more closely related to skeletal muscle mass than adiposity or BMI. HFpEF and HFrEF show distinct body composition abnormalities

Association Between EFT and BNP/NT-proBNP

A consistent positive correlation was observed between EFT and natriuretic peptide levels (BNP or NT-proBNP, depending on the study) across multiple populations. In CAD patients, Börekçi et al. [15] reported that EFT independently predicted elevated NT-proBNP levels, suggesting its role in identifying subclinical cardiac stress, defined as early myocardial strain without overt symptoms or functional impairment. Similarly, in HF patients, Tabakci et al. [16] found that higher EFT was associated with increased BNP levels and worse functional status (NYHA class), highlighting its prognostic value in symptomatic disease. Notably, the relationship between EFT and natriuretic peptides varied by HF phenotype. In HFpEF, van Woerden et al. [23] demonstrated that EFT correlated with biomarkers of myocardial injury (troponin T and creatine kinase-MB), implicating EAT as a mediator of metabolic-inflammatory injury. In contrast, in HFrEF, Selvaraj et al. [24] observed that NT-proBNP was more closely tied to muscle mass than adiposity, reflecting the prominence of sarcopenia-driven cardiac stress in this subgroup. A paradoxical finding emerged in nonischemic cardiomyopathy (NICMP), where Tabakci et al. [16] reported that lower EFT predicted higher BNP levels and worse NYHA class. This may reflect advanced disease severity, akin to cachexia, a catabolic state characterized by fat and muscle depletion, well-documented in chronic HF [23,24]. Mechanistically, EAT volume (assessed via imaging) and its bioactive properties (e.g., adipokine secretion) were distinct focuses across studies: Fosshaug et al. [20] analyzed EAT composition (e.g., interleukin-6, IL-6, expression), whereas others measured EFT thickness [15,16,23]. Yang et al. [21] scored lowest in quality assessment (NOS: 4/9) due to inadequate adjustment for confounders (e.g., metabolic comorbidities) and unclear exposure assessment, limiting the generalizability of their C1q/tumor necrosis factor-related protein 1 (CTRP1) findings. Importantly, EFT/BNP/NT-proBNP served dual roles: risk stratification in overt HF [16,23] and early detection in subclinical populations, e.g., CAD [15] and stroke [17].

EFT and BNP/NT-proBNP in Risk Stratification

EFT and BNP/NT-proBNP demonstrated prognostic value in HF and related conditions. In de novo HF [22], adiponectin and proBNP predicted readmission and mortality, with nutrient deprivation exacerbating adipokine dysregulation. In systolic HF [20], EAT’s inflammatory profile (elevated IL-6 and peroxisome proliferator-activated receptor alpha) and fatty acid composition were linked to NT-proBNP levels, implicating EAT in myocardial remodeling. For HFpEF [21], elevated adipokine CTRP1 in EAT and plasma was associated with IL-6-driven pathogenesis and worse outcomes.

Subgroup Variations: HF Phenotypes and Comorbidities

Differences emerged between HF subtypes. HFrEF patients [24] exhibited muscle loss as a stronger NT-proBNP predictor than adiposity. In contrast, HFpEF patients [23] had EFT linked to metabolic comorbidities, such as atrial fibrillation (AF) and type 2 diabetes mellitus (T2DM). In non-HF populations (e.g., obesity and stroke), EFT and NT-proBNP reflected early cardiovascular dysfunction [13,17] but were less predictive in asymptomatic obesity [14].

Risk of Bias Assessment Results

Among the 12 studies, four achieved high-quality ratings (NOS scores ≥7): Tabakci et al. [16] scored 9/9, demonstrating excellent selection, comparability, and outcome assessment; Temiz Karadag et al. [18], Fosshaug et al. [20], and van Woerden et al. [23] each scored 8/9, showing robust methodology with minor limitations in outcome measurement. Seven studies [13-15,17,19,22,24] scored 5-7/9, indicating moderate quality, primarily due to inadequate control for confounders (comparability domain) or potential selection bias. Yang et al. [21] scored 4/9, reflecting a high risk of bias from insufficient adjustment for key covariates and unclear exposure assessment. Overall, the majority of studies (11/12) met acceptable quality thresholds (NOS score ≥5), supporting the reliability of the synthesized evidence (Table 2).

**Table 2:** Risk of bias assessment using Newcastle-Ottawa Scale

Study	Selection (0-4)	Comparability (0-2)	Outcome/exposure (0-3)	Total score (out of 9)	Risk of bias
Bassareo et al. [13]	3	1	2	6	Moderate
Saritas et al. [14]	3	1	2	6	Moderate
Börekçi et al. [15]	3	1	2	6	Moderate
Tabakci et al. [16]	4	2	3	9	Low
Altun et al. [17]	3	1	2	6	Moderate
Temiz Karadag et al. [18]	4	2	2	8	Low
Karayannis et al. [19]	2	1	2	5	Moderate
Fosshaug et al. [20]	3	2	3	8	Low
Yang et al. [21]	2	1	1	4	High
Agra et al. [22]	3	2	2	7	Moderate
van Woerden et al. [23]	4	2	2	8	Low
Selvaraj et al. [24]	2	1	2	5	Moderate

Clinical and Mechanistic Insights Into EFT and BNP/NT-proBNP Associations

The reviewed studies revealed distinct patterns in the relationship between EFT and BNP/NT-proBNP across cardiovascular conditions. In HF subtypes, divergent associations were observed. Patients with HFrEF demonstrated stronger relationships between NT-proBNP and muscle mass than adiposity [24], suggesting sarcopenia rather than fat distribution drives NT-proBNP elevation in this population. In contrast, those with HFpEF showed significant correlations between EFT and metabolic comorbidities (AF and T2DM) as well as biomarkers of myocardial injury (troponin T) [23]. These findings highlight EFT's role as a metabolic-inflammatory driver in HFpEF, while NT-proBNP in HFrEF primarily reflects myocyte stress from volume overload.

A notable paradox emerged in studies of NICMP, where Tabakci et al. [16] reported that lower EFT correlated with higher BNP levels and worse NYHA functional class. This counterintuitive finding suggests that extreme fat depletion may indicate advanced disease severity in NICMP, potentially aligning with cachexia paradigms. However, this observation requires further validation in larger cohorts.

In populations without overt HF, EFT and NT-proBNP served as markers of subclinical cardiovascular dysfunction. Bassareo et al. [13] found that elevated EFT and NT-proBNP correlated with diastolic dysfunction in adolescents with Cushing's syndrome, while Altun et al. [17] demonstrated associations between increased EFT, NT-proBNP levels, and arterial stiffness in acute ischemic stroke patients. However, Saritas et al. [14] reported that while obese children had higher EFT and NT-proBNP levels than controls, these markers did not correlate with cardiac function, raising questions about their utility in asymptomatic metabolic risk assessment.

Therapeutic implications emerged from studies examining the bioactive properties of EAT. Fosshaug et al. [20] identified proinflammatory gene expression profiles in EAT from systolic HF patients, with a particular emphasis on IL-6 and fatty acid mediators that correlated with NT-proBNP levels. Similarly, Yang et al. [21] found that elevated levels of the adipokine CTRP1 in both EAT and plasma were associated with worse outcomes in congestive HF patients. Agra et al. [22] further demonstrated that nutrient starvation upregulated adiponectin expression in EAT, which independently predicted adverse outcomes in de novo HF patients. These findings collectively suggest that targeted interventions addressing EAT-derived inflammatory mediators or metabolic pathways may offer novel therapeutic approaches in HF management (Table 3).

**Table 3:** Clinical and mechanistic insights from EFT and BNP/NT-proBNP associations HFrEF: heart failure with reduced ejection fraction, NT-proBNP: N-terminal pro-B-type natriuretic peptide, HFpEF: heart failure with preserved ejection fraction, EFT: epicardial fat thickness, AF: atrial fibrillation, T2DM: type 2 diabetes mellitus, NYHA: New York Heart Association, BNP: B-type natriuretic peptide, NICMP: nonischemic cardiomyopathy, CAD: coronary artery disease, SYNTAX: synergy between PCI with Taxus and cardiac surgery, CRP: C-reactive protein, ESR: erythrocyte sedimentation rate, HF: heart failure, EAT: epicardial adipose tissue, proBNP: pro-B-type natriuretic peptide

Population	Key association	Clinical implications
HFrEF patients	NT-proBNP linked more to muscle mass than adiposity [24]	Sarcopenia, not fat distribution, drives NT-proBNP elevation and highlights need for nutritional/muscle interventions
HFpEF patients	EFT correlates with AF, T2DM, and myocardial injury biomarkers [23]	Suggests EFT as a marker of metabolic-inflammatory HFpEF phenotype; may guide comorbid disease management
Nonischemic cardiomyopathy	Lower EFT predicts worse NYHA class and higher BNP [16]	Paradoxical finding: reduced EFT may indicate advanced disease severity in NICMP
CAD patients	EFT independently predicts NT-proBNP and SYNTAX score [15]	Supports EFT as a surrogate for coronary complexity and subclinical cardiac stress
Obesity (pediatric/adult)	Elevated EFT/NT-proBNP in obesity, but no cardiac dysfunction [14]	Questions routine biomarker use in asymptomatic obesity; EFT may reflect metabolic risk, not HF
Systemic sclerosis	EFT correlates with inflammation (CRP, ESR) but not BNP [18]	Implicates EFT in subclinical cardiac involvement, independent of natriuretic peptides
Acute ischemic stroke	EFT correlates with NT-proBNP and aortic stiffness [17]	Suggests shared pathways between arterial dysfunction, EFT, and cardiac stress
De novo HF	Adiponectin (from EAT) and proBNP predict outcomes [22]	Nutrient starvation upregulates adiponectin, worsening prognosis; links metabolism to HF progression

Discussion

The findings of this systematic review provide compelling evidence for the complex interplay between EFT and BNP/NT-proBNP levels across various cardiovascular conditions, offering important insights into their combined role in HF risk stratification. Our analysis of 12 studies encompassing diverse populations reveals several key patterns that advance our understanding of the pathophysiological mechanisms linking EAT to cardiac dysfunction. The most consistent observation across studies was the significant correlation between increased EFT and elevated BNP/NT-proBNP levels in patients with established cardiovascular disease, particularly in HF populations [15,16,23]. This association appears to be mediated through multiple pathways, including mechanical effects of pericardial constraint, paracrine signaling from adipokines, and systemic inflammation, all of which contribute to myocardial stress and subsequent natriuretic peptide release.

The relationship between EFT and natriuretic peptides demonstrated important variations across different HF phenotypes. In HFrEF patients, the studies by Selvaraj et al. [24] and Karayannis et al. [19] revealed that NT-proBNP levels showed stronger associations with muscle wasting than with adiposity measures, suggesting that the neurohormonal activation characteristic of HFrEF may be more closely tied to catabolic processes than to epicardial fat deposition. This finding aligns with existing literature emphasizing the prognostic importance of sarcopenia in advanced HF [25]. Conversely, in HFpEF populations, the works of van Woerden et al. [23] and Yang et al. [21] demonstrated robust correlations between EFT and metabolic comorbidities, with EFT serving as a marker of the systemic proinflammatory state that characterizes this condition. These observations support the emerging paradigm of HFpEF as a metabolic-inflammatory disorder where epicardial fat may play a central role in disease pathogenesis through its endocrine functions [26].

The study by Tabakci et al. [16] presented a particularly intriguing finding regarding NICMP, where lower EFT paradoxically correlated with worse clinical status and higher BNP levels. This observation may reflect the advanced disease state in these patients, potentially indicating a cachexia-like phenomenon where excessive fat depletion signals poor prognosis. This aligns with previous reports in chronic HF populations where excessive fat and muscle loss correlate with disease severity [27]. The inverse relationship between EFT and disease severity in NICMP contrasts with most other studies in our review and highlights the need for phenotype-specific interpretation of EFT measurements.

In non-HF populations, the studies by Bassareo et al. [13] and Altun et al. [17] demonstrated that EFT and NT-proBNP elevations often precede overt cardiac dysfunction, suggesting their potential role in early risk stratification. The findings in adolescent Cushing's syndrome patients [13] were particularly noteworthy, showing that metabolic derangements can induce measurable changes in cardiac structure and function even in young populations. Similarly, the study of acute ischemic stroke patients [17] revealed associations between EFT, arterial stiffness, and NT-proBNP, supporting the concept of a shared pathophysiology between cardiac and cerebrovascular disease. However, the study by Saritas et al. [14] in obese children cautioned against overinterpretation of these markers in isolation, as they found no correlation between EFT/NT-proBNP and cardiac function in this population, suggesting that metabolic adaptations in childhood obesity may differ from those in adults.

The mechanistic insights from studies examining the biological properties of EAT provide compelling evidence for its active role in cardiovascular pathophysiology. Fosshaug et al. [20] demonstrated distinct proinflammatory gene expression profiles in the EAT of systolic HF patients, with particular elevation of IL-6 and other mediators known to promote myocardial remodeling. These findings complement previous work showing that epicardial fat exhibits a more pronounced inflammatory profile than other fat depots [28]. Similarly, Yang et al. [21] identified CTRP1 as a novel adipokine linking EAT dysfunction to HF progression through IL-6 and aldosterone pathway activation. The study by Agra et al. [22] further expanded our understanding by demonstrating nutrient-sensitive regulation of adiponectin in EAT and its independent prognostic value in de novo HF. These collective findings position EAT as not just a passive fat depot but as an active endocrine organ capable of modulating cardiac function through multiple pathways.

The clinical implications of these findings are significant for risk stratification and potential therapeutic targeting. The consistent association between EFT and adverse outcomes across multiple studies suggests that echocardiographic or CMR-based assessment of EFT could enhance existing risk prediction models, particularly in HFpEF, where traditional risk factors perform poorly [29]. The strong correlations between EFT and metabolic comorbidities in HFpEF [23] support the growing emphasis on metabolic interventions in this population. Furthermore, identifying specific adipokines and inflammatory mediators originating from EAT [20,21] opens potential avenues for targeted therapies aimed at modulating epicardial fat's endocrine functions.

Our findings must be interpreted in the context of several methodological considerations across the included studies. The majority of studies used echocardiography for EFT measurement, which, while practical and widely available, has recognized limitations in reproducibility compared to cardiac MRI [30]. The studies by van Woerden et al. [23] and Selvaraj et al. [24] utilizing CMR provided more precise volumetric assessments of epicardial fat but were limited to specialized centers. The timing and methods of BNP/NT-proBNP measurement also varied considerably across studies, with some using fasting levels and others random measurements, potentially affecting result comparability. Additionally, the cross-sectional design of most included studies precludes definitive conclusions about causality in the observed associations.

Compared to existing literature, our findings confirm and extend current knowledge about EFT and natriuretic peptides. The correlation between EFT and BNP/NT-proBNP aligns with previous reports in metabolic syndrome and CAD populations [31]. However, our systematic evaluation across diverse clinical conditions provides novel insights into how these relationships vary by disease state. The distinct patterns observed in HFrEF vs. HFpEF particularly contribute to the growing recognition of these as fundamentally different diseases with different pathophysiological drivers [32]. The identification of EFT as a stronger correlate of metabolic derangements in HFpEF supports recent calls for phenotype-specific management approaches [33].

Limitations

Several limitations of this review warrant consideration. First, the included studies exhibited heterogeneity in population characteristics, measurement techniques (e.g., echocardiography vs. cardiac MRI for EFT assessment, differing BNP/NT-proBNP assay protocols), and outcome definitions, precluding meta-analysis. Second, most studies were observational and cross-sectional in design, limiting causal inferences and obscuring the temporal sequence between EFT increases and natriuretic peptide elevation. Third, potential publication bias may exist, as small studies with null findings might not have been published. Fourth, the quality assessment revealed that several studies had a moderate risk of bias, particularly in controlling for confounders. Fifth, the predominance of case-control designs introduces potential selection bias that may affect generalizability. Finally, the absence of standardized EFT thresholds across studies complicates clinical interpretation and translation.

## Conclusions

This systematic review synthesizes observational evidence supporting the association between EFT and BNP/NT-proBNP levels in cardiovascular risk stratification across clinical contexts. The findings suggest distinct patterns in different HF phenotypes, with echocardiographic EFT measurements showing particular relevance in HFpEF through metabolic-inflammatory correlations. While demonstrating consistent prognostic value for imaging-assessed EFT, the review also highlights potential biological mechanisms involving EAT-derived mediators. Future research should prioritize the following: 1) standardized EFT measurement protocols, 2) longitudinal designs to clarify temporal relationships, and 3) targeted interventions including pharmacologic (e.g., anti-inflammatory) and metabolic strategies to modify EAT biology. These insights contribute to a more nuanced understanding of cardiac-adipose tissue interactions in risk assessment.

## Appendices

**Table 4:** Search strings

Database	Search string
PubMed/MEDLINE	(epicardial fat OR epicardial adipose tissue OR epicardial fat thickness) AND (BNP OR B-type natriuretic peptide OR NT-proBNP OR N-terminal pro-B-type natriuretic peptide) AND (heart failure OR cardiac failure) AND (risk stratification OR prognosis OR predictive value)
Embase	('epicardial fat':ti,ab,kw OR 'epicardial adipose tissue':ti,ab,kw OR 'epicardial fat thickness':ti,ab,kw) AND ('bnp':ti,ab,kw OR 'b type natriuretic peptide':ti,ab,kw OR 'nt probnp':ti,ab,kw OR 'n terminal pro b type natriuretic peptide':ti,ab,kw) AND ('heart failure':ti,ab,kw) AND ('risk stratification':ti,ab,kw OR 'prognosis':ti,ab,kw OR 'predictive value':ti,ab,kw)
Scopus	TITLE-ABS-KEY("epicardial fat" OR "epicardial adipose tissue" OR "epicardial fat thickness") AND TITLE-ABS-KEY("BNP" OR "B-type natriuretic peptide" OR "NT-proBNP" OR "N-terminal pro-B-type natriuretic peptide") AND TITLE-ABS-KEY("heart failure") AND TITLE-ABS-KEY("risk stratification" OR "prognosis" OR "predictive value")
Web of Science	TS=("epicardial fat" OR "epicardial adipose tissue" OR "epicardial fat thickness") AND TS=("BNP" OR "B-type natriuretic peptide" OR "NT-proBNP" OR "N-terminal pro-B-type natriuretic peptide") AND TS=("heart failure") AND TS=("risk stratification" OR "prognosis" OR "predictive value")
Cochrane Library	("epicardial fat" OR "epicardial adipose tissue" OR "epicardial fat thickness") AND ("BNP" OR "B-type natriuretic peptide" OR "NT-proBNP" OR "N-terminal pro-B-type natriuretic peptide") AND ("heart failure") AND ("risk stratification" OR "prognosis" OR "predictive value")
